# Neurofilament light levels predict clinical progression and death in multiple system atrophy

**DOI:** 10.1093/brain/awac253

**Published:** 2022-07-29

**Authors:** Viorica Chelban, Elham Nikram, Alexandra Perez-Soriano, Carlo Wilke, Alexandra Foubert-Samier, Nirosen Vijiaratnam, Tong Guo, Edwin Jabbari, Simisola Olufodun, Mariel Gonzalez, Konstantin Senkevich, Brice Laurens, Patrice Péran, Olivier Rascol, Anne Pavy Le Traon, Emily G Todd, Alyssa A Costantini, Sondos Alikhwan, Ambreen Tariq, Bai Lin Ng, Esteban Muñoz, Celia Painous, Yaroslau Compta, Carme Junque, Barbara Segura, Kristina Zhelcheska, Henny Wellington, Ludger Schöls, Zane Jaunmuktane, Christopher Kobylecki, Alistair Church, Michele T M Hu, James B Rowe, P Nigel Leigh, Luke Massey, David J Burn, Nicola Pavese, Tom Foltynie, Sofya Pchelina, Nicholas Wood, Amanda J Heslegrave, Henrik Zetterberg, Martina Bocchetta, Jonathan D Rohrer, Maria J Marti, Matthis Synofzik, Huw R Morris, Wassilios G Meissner, Henry Houlden

**Affiliations:** Department of Neuromuscular Diseases, Queen Square Institute of Neurology, University College London, London WC1N 3BG, UK; Neurobiology and Medical Genetics Laboratory, “Nicolae Testemitanu” State University of Medicine and Pharmacy, MD 2004 Chisinau, Republic of Moldova; Peninsula Technology Assessment Group (PenTAG), University of Exeter, Exeter EX 2LU, UK; Movement Disorders Unit, Neurology Service, Hospital Clínic de Barcelona, Barcelona 08036, Spain; Parkinson's Disease and Movement Disorders Unit, Neurology Department, Institute of Biomedical Research August Pi i Sunyer (IDIBAPS), Barcelona 08036, Spain; Parkinson's Disease and Movement Disorders Unit, Neurology Department, Centro de Investigación Biomédica en Red sobre Enfermedades Neurodegenerativas, Madrid 28029, Spain; Division Translational Genomics of Neurodegenerative Diseases, Hertie-Institute for Clinical Brain Research and Center of Neurology, University of Tübingen, 72074 Tübingen, Germany; German Center for Neurodegenerative Diseases (DZNE), 72074 Tübingen, Germany; CRMR AMS, Service de Neurologie – Maladies Neurodégénératives, CHU de Bordeaux, F-33000 Bordeaux, France; Université de Bordeaux, CNRS, IMN, UMR 5293, F-33000 Bordeaux, France; Université de Bordeaux, INSERM, BPH, U1219, F-33000 Bordeaux, France; Inserm, CIC 1401 Bordeaux, Clinical Epidemiology Unit, F-33000 Bordeaux, France; Department Clinical and Movement Neuroscience, UCL Queen Square Institute of Neurology, University College London, London WC1N 3BG, UK; Department Clinical and Movement Neuroscience, UCL Queen Square Institute of Neurology, University College London, London WC1N 3BG, UK; Department Clinical and Movement Neuroscience, UCL Queen Square Institute of Neurology, University College London, London WC1N 3BG, UK; Department of Neuromuscular Diseases, Queen Square Institute of Neurology, University College London, London WC1N 3BG, UK; Department of Neuromuscular Diseases, Queen Square Institute of Neurology, University College London, London WC1N 3BG, UK; Neurogenomics and Precision Medicine (NAP-Med) Laboratory, The Neuro (Montreal Neurological Institute-Hospital), Montreal, QC H3A 2B4, Canada; Department of Neurology & Neurosurgery, McGill University, Montreal, QC H3A 2B4, Canada; Laboratory of Human Genetics, Petersburg Nuclear Physics Institute named by B.P. Konstantinov of National Research Centre 'Kurchatov Institute', Gatchina 188300, Russia; Laboratory of Medical Genetics, Pavlov First Saint-Petersburg State Medical University, St. Petersburg 197022, Russia; CRMR AMS, Service de Neurologie – Maladies Neurodégénératives, CHU de Bordeaux, F-33000 Bordeaux, France; Université de Bordeaux, CNRS, IMN, UMR 5293, F-33000 Bordeaux, France; ToNIC, Toulouse NeuroImaging Center, UMR 1214, Université de Toulouse, 31024 Toulouse, France; CRMR AMS, CHU de Toulouse, 31300 Toulouse, France; Clinical Investigation Center CIC 1436, NS-Park/F-CRIN Network and NeuroToul COEN Center; Inserm, University of Toulouse 3 and CHU of Toulouse, F-31000 Toulouse, France; Departments of Neurosciences and Clinical Pharmacology, CHU Toulouse and University of Toulouse 3, F-31000 Toulouse, France; CRMR AMS, CHU de Toulouse, 31300 Toulouse, France; Institut des Maladies Métaboliques et Cardiovasculaires, Inserm U 1297, Toulouse University, F-31000 Toulouse, France; Dementia Research Centre, Department of Neurodegenerative Disease, UCL Queen Square Institute of Neurology, University College London, WC1N 3BG London, UK; Department Clinical and Movement Neuroscience, UCL Queen Square Institute of Neurology, University College London, London WC1N 3BG, UK; Department of Neuromuscular Diseases, Queen Square Institute of Neurology, University College London, London WC1N 3BG, UK; Department of Neuromuscular Diseases, Queen Square Institute of Neurology, University College London, London WC1N 3BG, UK; Department of Economics, University College London, London WC1N 3BG, UK; Movement Disorders Unit, Neurology Service, Hospital Clínic de Barcelona, Barcelona 08036, Spain; Parkinson's Disease and Movement Disorders Unit, Neurology Department, Institute of Biomedical Research August Pi i Sunyer (IDIBAPS), Barcelona 08036, Spain; Parkinson's Disease and Movement Disorders Unit, Neurology Department, Centro de Investigación Biomédica en Red sobre Enfermedades Neurodegenerativas, Madrid 28029, Spain; Movement Disorders Unit, Neurology Service, Hospital Clínic de Barcelona, Barcelona 08036, Spain; Parkinson's Disease and Movement Disorders Unit, Neurology Department, Institute of Biomedical Research August Pi i Sunyer (IDIBAPS), Barcelona 08036, Spain; Parkinson's Disease and Movement Disorders Unit, Neurology Department, Centro de Investigación Biomédica en Red sobre Enfermedades Neurodegenerativas, Madrid 28029, Spain; Movement Disorders Unit, Neurology Service, Hospital Clínic de Barcelona, Barcelona 08036, Spain; Parkinson's Disease and Movement Disorders Unit, Neurology Department, Institute of Biomedical Research August Pi i Sunyer (IDIBAPS), Barcelona 08036, Spain; Parkinson's Disease and Movement Disorders Unit, Neurology Department, Centro de Investigación Biomédica en Red sobre Enfermedades Neurodegenerativas, Madrid 28029, Spain; Parkinson's Disease and Movement Disorders Unit, Neurology Department, Institute of Biomedical Research August Pi i Sunyer (IDIBAPS), Barcelona 08036, Spain; Parkinson's Disease and Movement Disorders Unit, Neurology Department, Centro de Investigación Biomédica en Red sobre Enfermedades Neurodegenerativas, Madrid 28029, Spain; Medical Psychology Unit, Department of Medicine, Institute of Neuroscience, University of Barcelona, 08035 Barcelona, Spain; Parkinson's Disease and Movement Disorders Unit, Neurology Department, Institute of Biomedical Research August Pi i Sunyer (IDIBAPS), Barcelona 08036, Spain; Parkinson's Disease and Movement Disorders Unit, Neurology Department, Centro de Investigación Biomédica en Red sobre Enfermedades Neurodegenerativas, Madrid 28029, Spain; Medical Psychology Unit, Department of Medicine, Institute of Neuroscience, University of Barcelona, 08035 Barcelona, Spain; Department of Neuromuscular Diseases, Queen Square Institute of Neurology, University College London, London WC1N 3BG, UK; Biomarkers Factory Laboratory, UK Dementia Research Institute, UCL Queen Square Institute of Neurology, London WC1N 3BG, UK; Division Translational Genomics of Neurodegenerative Diseases, Hertie-Institute for Clinical Brain Research and Center of Neurology, University of Tübingen, 72074 Tübingen, Germany; German Center for Neurodegenerative Diseases (DZNE), 72074 Tübingen, Germany; Queen Square Brain Bank for Neurological Disorders, UCL Queen Square Institute of Neurology, WC1N 3BG London, UK; Department of Neurology, Manchester Academic Health Science Centre, Northern Care Alliance NHS Foundation Trust, Stott Lane, Salford M6 8HD, UK; Division of Neuroscience and Experimental Psychology, School of Biological Sciences, University of Manchester, Oxford Road, Manchester M13 9PT, UK; Department of Neurology, Royal Gwent Hospital, Newport NP20 2UB, UK; Division of Neurology, Nuffield Department of Clinical Neurosciences, University of Oxford, Oxford OX3 9DU, UK; Department of Clinical Neurosciences, Cambridge University, Cambridge CB3 0SZ, UK; MRC Cognition and Brain Sciences Unit, University of Cambridge, CB3 0SZ Cambridge, UK; Neurology Department, Cambridge University Hospitals NHS Trust, Cambridge CB2 0QQ, UK; Department of Neuroscience, Brighton and Sussex Medical School, Brighton BN1 9PX, UK; Neurology Department, University Hospitals Dorset, Poole BH15 2JB, UK; Faculty of Medical Sciences, Clinical Ageing Research Unit, Newcastle University, NE4 5PL Newcastle, UK; Department of Neurodegenerative Disease, UCL Institute of Neurology, Queen Square, WC1N 3BG London, UK; Department Clinical and Movement Neuroscience, UCL Queen Square Institute of Neurology, University College London, London WC1N 3BG, UK; Laboratory of Human Genetics, Petersburg Nuclear Physics Institute named by B.P. Konstantinov of National Research Centre 'Kurchatov Institute', Gatchina 188300, Russia; Laboratory of Medical Genetics, Pavlov First Saint-Petersburg State Medical University, St. Petersburg 197022, Russia; Department Clinical and Movement Neuroscience, UCL Queen Square Institute of Neurology, University College London, London WC1N 3BG, UK; Biomarkers Factory Laboratory, UK Dementia Research Institute, UCL Queen Square Institute of Neurology, London WC1N 3BG, UK; Biomarkers Factory Laboratory, UK Dementia Research Institute, UCL Queen Square Institute of Neurology, London WC1N 3BG, UK; Department of Neurodegenerative Disease, UCL Institute of Neurology, Queen Square, WC1N 3BG London, UK; Department of Psychiatry and Neurochemistry, Institute of Neuroscience and Physiology, the Sahlgrenska Academy at the University of Gothenburg, 405 30 Mölndal, Sweden; Clinical Neurochemistry Laboratory, Sahlgrenska University Hospital, 405 30 Mölndal, Sweden; Hong Kong Center for Neurodegenerative Diseases, Clear Water Bay, Hong Kong 1512-1518, China; Dementia Research Centre, Department of Neurodegenerative Disease, UCL Queen Square Institute of Neurology, University College London, WC1N 3BG London, UK; Dementia Research Centre, Department of Neurodegenerative Disease, UCL Queen Square Institute of Neurology, University College London, WC1N 3BG London, UK; Movement Disorders Unit, Neurology Service, Hospital Clínic de Barcelona, Barcelona 08036, Spain; Parkinson's Disease and Movement Disorders Unit, Neurology Department, Institute of Biomedical Research August Pi i Sunyer (IDIBAPS), Barcelona 08036, Spain; Parkinson's Disease and Movement Disorders Unit, Neurology Department, Centro de Investigación Biomédica en Red sobre Enfermedades Neurodegenerativas, Madrid 28029, Spain; Division Translational Genomics of Neurodegenerative Diseases, Hertie-Institute for Clinical Brain Research and Center of Neurology, University of Tübingen, 72074 Tübingen, Germany; German Center for Neurodegenerative Diseases (DZNE), 72074 Tübingen, Germany; Department Clinical and Movement Neuroscience, UCL Queen Square Institute of Neurology, University College London, London WC1N 3BG, UK; CRMR AMS, Service de Neurologie – Maladies Neurodégénératives, CHU de Bordeaux, F-33000 Bordeaux, France; Université de Bordeaux, CNRS, IMN, UMR 5293, F-33000 Bordeaux, France; Department of Medicine, University of Otago, Christchurch 8140, New Zealand; New Zealand Brain Research Institute, Christchurch 8011, New Zealand; Department of Neuromuscular Diseases, Queen Square Institute of Neurology, University College London, London WC1N 3BG, UK

**Keywords:** multiple system atrophy, MSA, NfL

## Abstract

Disease-modifying treatments are currently being trialled in multiple system atrophy. Approaches based solely on clinical measures are challenged by heterogeneity of phenotype and pathogenic complexity. Neurofilament light chain protein has been explored as a reliable biomarker in several neurodegenerative disorders but data on multiple system atrophy have been limited. Therefore, neurofilament light chain is not yet routinely used as an outcome measure in multiple system atrophy. We aimed to comprehensively investigate the role and dynamics of neurofilament light chain in multiple system atrophy combined with cross-sectional and longitudinal clinical and imaging scales and for subject trial selection.

In this cohort study, we recruited cross-sectional and longitudinal cases in a multicentre European set-up. Plasma and CSF neurofilament light chain concentrations were measured at baseline from 212 multiple system atrophy cases, annually for a mean period of 2 years in 44 multiple system atrophy patients in conjunction with clinical, neuropsychological and MRI brain assessments. Baseline neurofilament light chain characteristics were compared between groups. Cox regression was used to assess survival; receiver operating characteristic analysis to assess the ability of neurofilament light chain to distinguish between multiple system atrophy patients and healthy controls. Multivariate linear mixed-effects models were used to analyse longitudinal neurofilament light chain changes and correlated with clinical and imaging parameters. Polynomial models were used to determine the differential trajectories of neurofilament light chain in multiple system atrophy. We estimated sample sizes for trials aiming to decrease neurofilament light chain levels.

We show that in multiple system atrophy, baseline plasma neurofilament light chain levels were better predictors of clinical progression, survival and degree of brain atrophy than the neurofilament light chain rate of change. Comparative analysis of multiple system atrophy progression over the course of disease, using plasma neurofilament light chain and clinical rating scales, indicated that neurofilament light chain levels rise as the motor symptoms progress, followed by deceleration in advanced stages. Sample size prediction suggested that significantly lower trial participant numbers would be needed to demonstrate treatment effects when incorporating plasma neurofilament light chain values into multiple system atrophy clinical trials in comparison to clinical measures alone.

In conclusion, neurofilament light chain correlates with clinical disease severity, progression and prognosis in multiple system atrophy. Combined with clinical and imaging analysis, neurofilament light chain can inform patient stratification and serve as a reliable biomarker of treatment response in future multiple system atrophy trials of putative disease-modifying agents.

## Teaser

In this large multiple system atrophy cohort, Chelban *et al.* show that plasma NfL correlates with clinical disease severity, progression and prognosis, and could help inform patient stratification and monitor treatment responses in future trials of putative disease-modifying agents.

## Introduction

Multiple system atrophy (MSA) is a rapidly progressive neurodegenerative disorder with an estimated prevalence of 4–5 per 100 000 individuals in Europe and North America. The condition manifests with a combination of cerebellar ataxia, parkinsonism and autonomic dysfunction.^1–4^ Fulfilment of clinical criteria is often only achieved in the later stages of the disease, thus offering limited opportunities for intervention.^5^ Furthermore, reliance on crude clinical rating scales and patient-reported outcome measures may reduce sensitivity to the efficacy of experimental medicines. Reliable, quantifiable biomarkers of neurodegeneration to complement clinical diagnosis in the early stages of the disease, to better monitor disease progression, and therapeutic responses, are therefore critical for future clinical trials exploring disease-modifying or neuroprotective agents.

Over the past two decades, CSF and blood neurofilament light chain (NfL) have been shown to be reliable biomarkers of axonal damage across a variety of neurological disorders.^6^ In axonal injury or during neurodegeneration, NfL is released into the interstitial fluid; consequently, the NfL levels in CSF and blood increase,^7^ thus making it possible to easily and repeatedly measure NfL for monitoring neuronal decay in the disease course.^8^ Despite NfL being extensively validated as a robust and reliable biomarker for assessing the severity and rate of progression of axonal degeneration, no studies have assessed the NfL profile and its prognostic value in MSA cohorts. However, smaller studies suggest that NfL might successfully enhance MSA clinical trials.^9–12^

Here we aimed to examine how blood and CSF NfL concentrations reflect disease severity and progression in MSA by combining clinical scores, regional brain atrophy rate and NfL levels in a large, multicentre, longitudinal cohort to help inform the design, patient stratification at baseline and monitor treatment response in future MSA clinical trials.

## Materials and methods

### Subjects

We recruited 212 patients with MSA and 40 age-matched healthy controls (HC) from MSA specialist centres in the UK (PROSPECT-M study), France, Spain, Germany and Russia. From the 212 patients included in the cross-sectional study arm, 44 had longitudinal follow-up (Supplementary Fig. 1A).

Plasma samples were obtained from 212 MSA patients. CSF samples were obtained from 114 MSA patients. Paired plasma and CSF samples were obtained in 105 MSA cases and 36 HC. Plasma NfL and MRI data from the same visit were available in 55 MSA patients and 42 HC. Controls were healthy partners of the patients. MSA subjects and HC did not differ significantly in age or sex.

The PROSPECT-M-UK study was approved by the London – Queen Square Research Ethics Committee (14/LO/1575); Pavlov First Saint-Petersburg State Medical University Ethics Committee (no. 204 dated 26 February 2018) and HCB/2015/0798.

### Clinical and neuroimaging assessments

MSA diagnosis was established following current diagnostic criteria.^13^ Patients were divided into MSA-parkinsonism (MSA-P) or MSA-cerebellar (MSA-C) subtypes depending on the predominant phenotype at recruitment. At each visit, patient’s neurological history, clinical signs, cognitive and functional status was assessed using the Unified MSA Rating Scale (UMSARS),^14^ Montreal Cognitive Assessment (MoCA)^15^ and Addenbrooke’s Cognitive Examination revised (ACE-R).^16^ A subset of participants also had volumetric T_1_-weighted 3 T MRI. Longitudinal cohort participants were assessed annually with the same standardized clinical assessments.

### Neurofilament light chain measurements

Plasma and CSF NfL concentrations were measured in duplicates using the 1-plex single molecule array (Simoa) kit (NF-Light®, Quanterix) on a Simoa HD-1 Analyzer (Quanterix)^17^ according to the manufacturer’s instructions. All blood and CSF samples from collaborating centres were tested in the UCL laboratory, using one batch of reagents (lot 501769). All NfL values were within the linear range of the assay. For plasma, the mean intra-assay coefficient of variation (CV) of duplicate determinations for concentration was 4.5%. In the CSF, the mean intra-assay CV was 3.8%.

### Statistical analysis

Clinical and imaging data were paired with NfL concentrations in plasma at each visit with MRI performed within 4 weeks from biofluid collection. Values are reported as mean ± SD and median and interquartile range (IQR).

This exploratory study had multiple aims and did not establish a single sample size calculation in advance. Nonetheless, research in similar neurodegenerative conditions show that 14–35 participants per study arm detected cross-sectional differences in NfL levels between cases and controls as outcome measures.^18,19^ Therefore, in this study, going beyond the necessary sample size, we used all available samples.

The threshold for statistical significance in all analyses was *P* < 0.05. The analysis was carried out using STATA v.14 (Stata Statistical Software: College Station, TX, USA: StataCorp LP), SPSS v.26 (SPSS Inc., Chicago, IL, USA) and R Studio (R-3.6.3). Detailed clinical, neuroimaging and statistical analysis can be found in the Supplementary material.

### Data availability

The data that support the findings of this study are available from the corresponding author, upon reasonable request.

## Results

### Subjects and clinical characteristics

In total, 212 patients with MSA and 40 age-matched HC from MSA specialist centres from five European countries were included in this study (Supplementary Fig. 1 and Table 1). The demographic and baseline clinical characteristics are presented in Table 1. The mean age at inclusion for MSA cases and HC was 64 years, ±0.6 years (range 41–87) and 65 years, ±1 years (range 43–86), respectively, with no significant sex differences between cases and controls. The average age of MSA onset was 58.2 years (±8.3 years, range 37–83).

**Table 1:** Subject characteristic for MSA patients included in the biomarkers study

	MSA-C	MSA-P	*P*-value	Controls
**Total number**	106	106		40
Sex, *n* female (%)	44 (44%)	56 (56%)	0.09	20 (50%)
Age at onset, years, median (range) [IQR]	58 (38–77) [51–63]	59 (37–83) [53–64]	0.75	Not available
Age at sample collection, years, median (range) [IQR]	64 (46–80) [56–69]	64 (41–87) [58–69]	0.69	64.5 (43–76) [59–68]
**Predominant symptom at onset, *n* (%)**			<0.001	
Autonomic failure	43 (58.1)	41 (56.3)		
Cerebellar syndrome	30 (40.5)	4 (5.6)		
Parkinsonian syndrome	1 (1.1)	26 (36.6)		
Other	0 (0)	1 (1.4)		
**Diagnostic certainty at last visit, *n* (%)**			0.109	
Possible MSA	27 (25.5)	15 (14.2)		
Probable MSA	70 (66.0)	82 (77.3)		
Definite MSA	9 (8.5)	9 (8.5)		
**Disease severity at baseline**				
UMSARS total (part I and II) (mean)	45.4 (16.5)	46.6 (15.9)	0.622	
**Disease milestones (baseline measures), *n* (%)**				
Minimal motor symptoms	18 (17.3)	16 (15.1)	0.16	
Postural instability	30 (28.6)	25 (22.6)	0.02	
Wheelchair-bound	16 (12.2)	18 (8.5)	0.07	
PEG recommended/speech loss	12 (5.7)	7 (3.3)	0.01	
Dependent on all daily activities	42 (19.9)	28 (13.2)	<0.001	
**Biomarkers (number of samples per MSA-type group)**				
Cross-sectional assessment (168 total number of cases), *n* (%)	75 (70.5)	92 (84.9)		
Longitudinal follow-up (44 total number of cases), *n* (%)	31 (29.5)	16 (15.1)		
Plasma and CSF matched samples (105 total number of cases), *n* (%)	50 (47.6)	55 (52.4)		
MRI and plasma matches, 55 at baseline (14 at follow-up), *n* (%)	32 (29.1)	29 (27.4)		

Most MSA cases (91.5%) had a diagnosis of probable (72%) or became definite MSA since recruitment (19.5%). Apart from cerebellar versus parkinsonian phenotype, there were no significant differences in clinical characteristics between MSA-P (*n* = 106) and MSA-C (*n* = 106) cases (Table 1). The MSA group was representative of a full range of disease severity with 30.6% of MSA patients (*n* = 65) recruited early in the disease (up to 3 years from disease onset).

Median disease duration at biofluid collection in MSA cases was 5 years (IQR 3–6.5 years). At the time of data analysis, 147 MSA patients (69.3%) were still alive and 81 of them continued to be followed up clinically after fluid collection. Since last fluid collection, 65 cases (30.7%) had died and 53 of them were followed up clinically from fluid collection until death. Autopsy was obtained in 18 of the deceased cases (27.7%) with MSA diagnosis confirmed in all these cases. The median clinical follow-up of living patients after biofluid collection was 2 years (range 1–8, IQR 1–3 years), while median survival of deceased patients after biofluid collection was 2.5 years (range 2–11 years). From the 212 MSA participants with baseline plasma, 44 participants returned for at least one and maximally six follow-up visits, with a mean number of 2.1 visits and a median observation time of 1 year from the baseline visit.

### Fluid biomarkers

The median concentration of NfL was significantly higher in MSA patients compared to HC for both the CSF (4329 pg/ml, IQR 2577–5862 versus 560 pg/ml, IQR 420–855, *P* < 0.001) and for plasma (39.9 pg/ml, IQR 27–48 versus 9.1 pg/ml, IQR 8.7–9.8, *P* < 0.001). Plasma NfL was positively associated with age at sample collection in MSA patients (rho = 0.21, *P* = 0.01) and HC. As NfL levels increase with age even in HC^6^ our results in the MSA cohort remained statistically significant after adjusting for age at sample collection (*P* < 0.001). There was no statistically significant linear correlation between plasma NfL levels and disease duration. No significant differences in plasma (*P* = 0.46) or CSF (*P* = 0.64) NfL levels was noted between MSA-P and MSA-C subgroups.

### Plasma versus CSF NfL, stratified by disease duration

There was only a moderate correlation between NfL levels in matched plasma and CSF samples (*n* = 105) (rho = 0.40, *P* < 0.001 in MSA cases, Supplementary Fig. 2A). In HC group we found a higher correlation between the two biofluids (rho = 0.70 *P* < 0.001) (Supplementary Fig. 2B). Recent studies in neurodegenerative conditions suggested that CSF NfL increased faster than plasma in the early stages of disease.^20^ We therefore assessed the CSF-plasma correlation, per disease stage. We stratified the MSA cases in early disease group (<3 years from onset), established disease (between 3 and 7 years) and late disease (>7 years of disease duration). There was no plasma-CSF correlation in the early stages (rho = 0.03, *P* = 0.8, *n* = 40). A correlation emerged as symptoms became more established (rho = −0.44, *P* < 0.001, *n* = 45) and strengthened as the disease progressed, with a very strong correlation, similar to HC, towards the late stages of MSA (rho = 0.68, *P* < 0.001, *n* = 20) (Supplementary Fig. 2C). Furthermore, in the early stages of disease we found a higher-fold change in CSF compared to plasma NfL (mean concentration in CSF was 6.9-fold higher and the mean plasma NfL was 4.1-fold higher in early MSA subjects compared to HC), probably accounting for the weak correlation. In later disease stages, we show similar fold changes in both plasma and CSF (Supplementary Fig. 2D), in keeping with the stronger correlation between CSF and plasma-derived NfL in this group. Given the association between plasma and CSF NfL, and the obvious advantage of a less-invasive blood biomarker, we focus on plasma NfL for subsequent analyses.

### NfL and MSA severity

**Figure 1:**
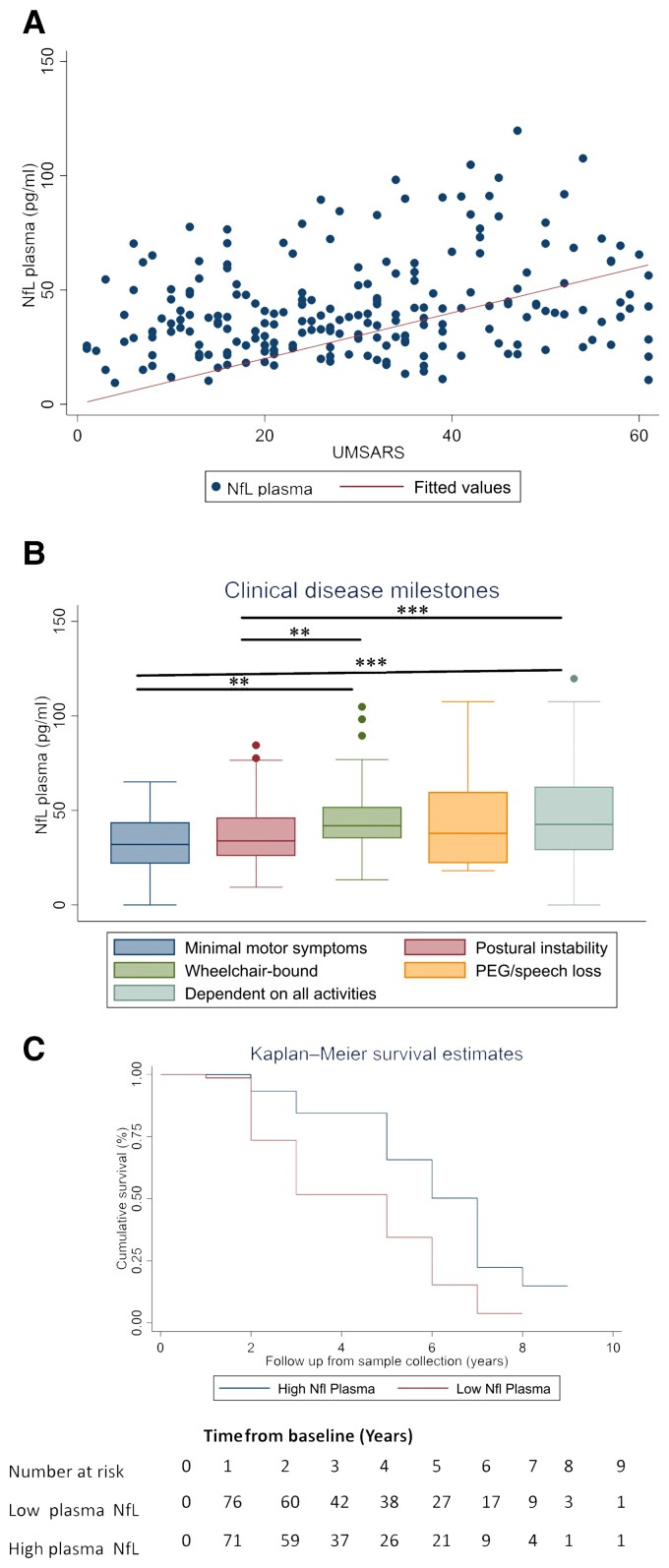
**NfL concentrations in patients with MSA correlate with disease severity and survival**. (**A**) Associations between NfL concentrations in plasma at baseline and cross-sectional clinical measure of UMSARS in MSA cases (*rho* = 0.24, *P* < 0.001). (**B**) NfL concentrations in plasma and disease stages defined by the disease milestones. Baseline plasma NfL concentrations by disease stage. Boxes show first and third quartiles, the central band shows the median, and the whiskers show data within 1.5 IQR of the median. The dots represent outliers. ***P < 0.001. (**C**) Higher plasma NfL values [blood NfL values above median (>38.1 pg/ml)] were associated with a more rapid progression and shorter overall survival (95% CI 1.43–3.80, HR 2.35, *P* = 0.001) when comparing the Kaplan–Meier curve of NfL levels stratified by low plasma NfL levels (<38.1 pg/ml).

We found a statistically significant correlation between plasma NfL levels at baseline (rho = 0.24, *P* < 0.001; Fig. 1A) and disease severity represented by the UMSARS. This remained statistically significant after adjustment for age (*P* = 0.001). Additionally, NfL concentrations differed significantly with increasing disease stages as defined by disease milestones (Fig. 1B).

### NfL and survival in MSA

Higher plasma NfL values at baseline were associated with a more rapid progression and shorter overall survival [*P* < 0.001, hazard ratio (HR) 1.02 (95% confidence interval (CI) 1.01–1.03), *n* = 194]. This survival predictive effect was consistent for both MSA subtypes (MSA-C HR 1.03, 95% CI 1.01–1.06, *P* = 0.003 and MSA-P HR 1.01, 95% CI 1.00–1.02, *P* = 0.005). Patients with blood NfL values above the median (>38.1 pg/ml) had an increased mortality rate [HR 2.35 (95% CI 1.41–3.90, *P* = 0.001) (Fig. 1C)]. Conversely, patients with the lowest plasma NfL tertile values had a lower mortality rate in comparison to the highest tertile (14.3% versus 44.3%, *P* < 0.001) while also having a longer survival time (6.0 versus 3.0 years, *P* < 0.001) (Supplementary Fig. 3A).

### Association of baseline plasma NfL with imaging measures

**Figure 2:**
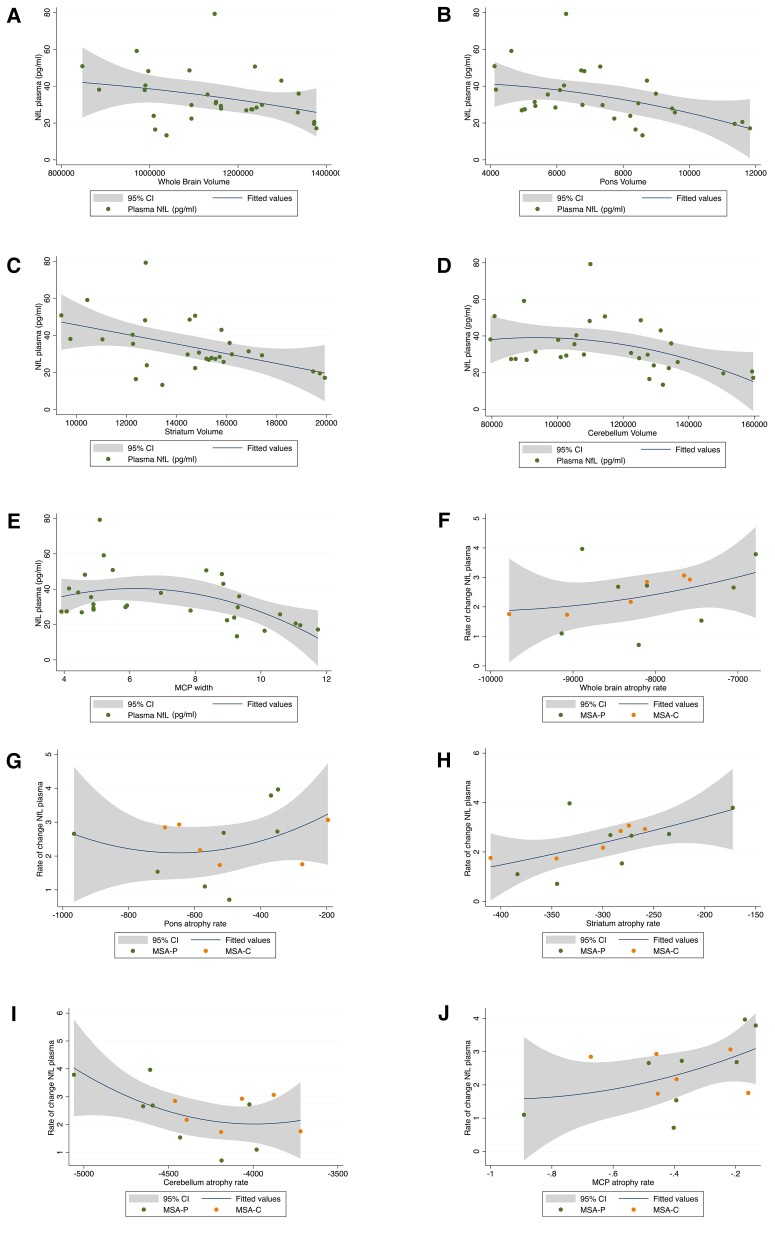
**Significant correlations between plasma NfL levels and neuroimaging outcomes.** (**A**–**E**) Associations between baseline plasma NfL levels with raw global and regional brain volumes and MCP width. (**F–J**) Associations between yearly NfL rate of changes and yearly brain atrophy rate of changes in MSA.

We assessed whether there was an association between baseline brain volume and plasma NfL in all MSA cases (*n* = 50). We found the strongest correlation in the striatum (rho = −0.56, *P* < 0.001), followed by the middle cerebellar peduncle (MCP) (rho = −0.31, *P* < 0.03). These results were further validated in a linear mixed-effects model (LMEM) analysis between NfL levels and regional brain volumes with adjustment for age, UMSARS and disease duration (absent collinearity, variance inflation factor < 1) where a statistically significant correlation between plasma NfL and striatum volume (rho = −0.34, *P* < 0.001) and modest statistically significant correlations with cerebellum, whole brain volume and pons were found (Table 2 and Fig. 2A–E). Furthermore, plasma NfL correlations with regional brain volumes were stronger when assessed by clinical subgroups. The strongest statistically significant correlations were found with the striatum in both MSA-C (*n* = 24) and MSA-P (*n* = 26) (MSA-C: rho = 0.48, *P* < 0.001, MSA-P: rho = 0.69, *P* < 0.001) and with cerebellum, in MSA-C (rho = 0.34, *P* < 0.01) (Supplementary Fig. 1 and Table 2).

**Table 2:** Brain regions and NfL correlations in MSA

Cross-sectional cases (*n* = 50)	Correlation		Multivariate regression	
Brain region	rho	*P*	Adj. *R*^2^	*P*
Whole brain	−0.26	0.06	**0**.**15**	**0**.**04**^a^
Pons	−0.17	0.214	**0**.**11**	**0**.**05**^a^
Striatum	**−0**.**56**	**<0**.**001**	**0**.**4**	**<0**.**001**^a^
Caudate subregion	**−0**.**5**	**<0**.**001**	**0**.**23**	**<0**.**001**^a^
Putamen subregion	**−0**.**5**	**<0**.**001**	**0**.**23**	**0**.**001**^a^
Cerebellum	−0.15	0.28	**0**.**16**	**0**.**01**^a^
MCP width	**−0**.**31**	**0**.**03**	0.02	0.24
**Longitudinal cases (*n* = 14)**			**Standardized β**	
**Brain region**				
Whole brain	**−0**.**390**	**0**.**027**	−0.225	0.122
Pons	**−0**.**560**	**<0**.**001**^a^	−0.484	**0**.**004**^a^
Striatum	**−0**.**536**	**0**.**002**^a^	−0.425	**0**.**006**^a^
Caudate subregion	**−0**.**53**	**0**.**001**^a^	−47.91	**0**.**002**^a^
Putamen subregion	**−0**.**45**	**0**.**007**^a^	−30.01	**0**.**018**^a^
Cerebellum	**−0**.**535**	**0**.**002**^a^	−0.444	**0**.**009**^a^
MCP width	**−0**.**594**	**<0**.**001**^a^	−0.555	**0**.**002**^a^
**LMEM rate of change**	**Est. FE**	**SE**	***R*²**	***P***
**Brain region**				
Whole brain	1277.8	3013.2	0.15	0.67
Pons	184.3	134.3	0.05	0.2
Striatum	95.28	92.83	0.39	0.32
Caudate subregion	80.23	60.83	**0**.**59**	**0**.**02**^a^
Putamen subregion	12.759	50.91	0.24	0.39
Cerebellum	737.7	1044.09	0.24	0.48
MCP width	0.34	0.16	**0**.**23**	**0**.**04**^a^

Correlation analysis was performed by Spearman rank correlation, from which we obtained *P* and rho for the association between these brain areas and NfL levels in all MSA cases at baseline. The multivariate regression analysis was performed adjusting for disease duration, UMSARS scores and age at sample to obtain β (the slope), the correlation value with adjusted *R*^2^ and *P*-value. LMEM analysis in the longitudinal cohort (*n* = 14, total number of scans = 33) was performed adjusting for disease duration, UMSARS and age at sample collection. Statistical significance was set at *P* < 0.05 and Bonferroni correction for multiple testing was used. An LMEM was also performed to determine the relationship between longitudinal brain volume rate of change and the extracted rate of plasma NfL change over time. Rates of change were extracted from LMEMs, using time (in years) as a fixed effect. Random slopes and intercepts of time per participant were included to produce individual regression coefficients per participant as previously described.^21^ Statistically significant values are in bold. Est.FE = estimated fixed effects; SE = standard error.

a Survived Bonferroni correction for multiple testing.

### NfL capturing longitudinal dynamics in MSA

Longitudinally, higher levels of baseline plasma NfL were significantly associated (*P* < 0.05) with greater subsequent decline in whole brain, pons, striatum, cerebellum volume and MCP width (Table 2). Using the rate of change in NfL and rate of change in brain region volumes in a longitudinal cohort of 14 patients, a LMEM analysis with adjustment for age and disease duration (Fig. 2F–J) revealed a statistically significant relationship between the rate of plasma NfL concentration with MCP width (rho = 0.23, *P* = 0.04). In addition, we assessed the usefulness of baseline plasma NfL in predicting yearly changes in UMSARS scores using LMEMs. Despite the small sample size (*n* = 44), we noted a positive relationship between baseline plasma NfL and subsequent UMSARS rate of change (*rho* = 0.26, *P* = 0.06) (Supplementary Fig. 3B). We found no significant associations between plasma NfL concentration at baseline and subsequent decline in cognition over a 2-year follow-up.

### Modelling of MSA progression using plasma NfL and UMSARS

**Figure 3:**
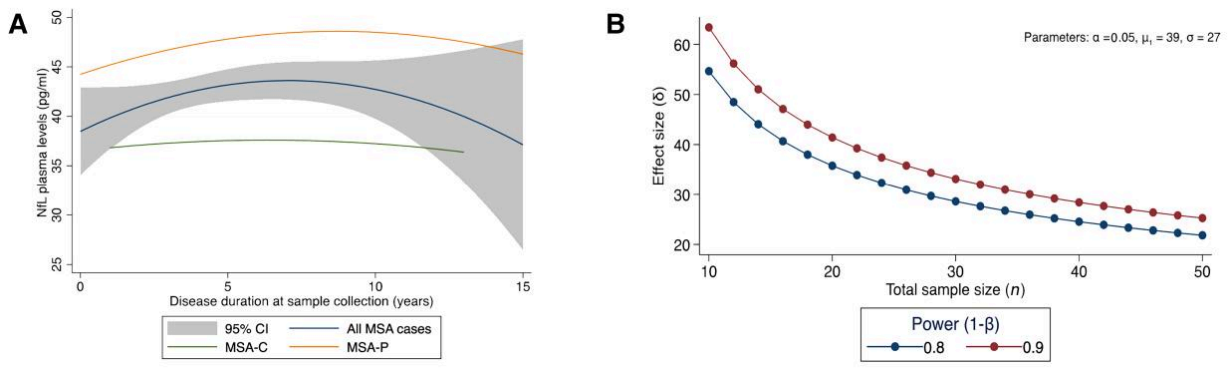
**NfL trajectories in MSA disease and sample size estimates for clinical trials**. (**A**) Association between NfL concentration in plasma, age, sex, MSA clinical subtype, severity, modelled with a polynomial function of age, disease duration, disease severity, their squares and their interactions in 212 MSA cases. The lines show a quadratic fit for all participants (*middle* line) with 95% CI (highlighted area), MSA-P only (*top* line), MSA-C only (*bottom* line). In the early disease stage, an increase in disease duration was associated with an increase in NfL concentration in plasma followed by a short plateau. At ∼7 years of disease duration, a decrease in NfL levels is observed. (**B**) Sample size estimates in MSA clinical trials. Required sample size per group to achieve 80 and 90% power implementing the reduction in plasma NfL as an outcome measure.

Given that plasma NfL showed a significant correlation with MSA severity but not with disease duration, we established a disease trajectory for MSA using plasma NfL and UMSARS scores to study disease progression over time. We found a positive association between higher plasma NfL and subsequent increase in UMSARS in the early stages of MSA (*P* < 0.001) (Supplementary Fig. 3C). In contrast, overall, a weaker correlation was noted in the later stages of MSA. This finding was re-affirmed when modelling MSA disease trajectories using baseline data (*n* = 212). Using a two-degree polynomial regression analysis on UMSARS and disease duration to model MSA progression (*r*^2^ = 0.20), we show that plasma NfL concentrations increased slowly initially, then accelerated with the progression of motor impairment, followed by a deceleration later in the disease (Fig. 3A). We then assessed whether this late deceleration of NfL was related to disease severity as defined by UMSARS tertiles. In the 42 cases with disease duration of 8 years (when the deceleration in NfL levels was noticed), over half of the cases had severe disease (UMSARS I + II >54, *n* = 22), 17 cases had moderate severity (UMSARS I + II 37-53) and only three MSA cases were classified as mild severity (UMSARS I + II <36). Using UMSARS IV, most of these cases (33 of 42) had severe disease (score of 4 or 5, very dependent or totally dependent/bedridden), seven cases had a score of 3 (more dependent, needs help with half of chores) and only two cases had a score of 2 (not completely independent, needs help with some chores).

### Exploring NfL as an outcome parameter in MSA trials

Finally, we performed sample size estimates for MSA intervention trials using the reduction of NfL levels as an outcome measure. Required sample sizes for interventional trials with 1-year follow-up were estimated on the basis of 12-month changes in plasma NfL. Estimated sample sizes per group to achieve 80 and 90% power with effect sizes ranging from 20 to 80%. We estimated that a trial with 28 participants (14 per group) would be able to detect a 30% reduction in annual NfL level at 80% power (Fig. 3B shows a range of other possible treatment effect sizes).

## Discussion

In this large and well-characterized MSA cohort we found that NfL correlates with disease severity particularly in the earlier stages of disease, with a curvilinear change as the disease progresses, and predicts survival. As NfL is increased in many neurodegenerative diseases, including several multisystemic ataxias^19^ and atypical parkinsonian conditions,^9^ the biomarker value of NfL levels lie in their ability to reflect disease severity and progression and in their potential as stratification biomarkers for MSA drug trials. Here we demonstrated that NfL levels correlated with cross-sectional disease severity, and showed a trending association with the longitudinal annual change of the UMSARS score within individuals, similarly with findings from a recent study with 1-year follow-up.^22^ The significant association we found between plasma NfL concentrations and MSA disease milestones further establishes an NfL-functional impairment profile over the disease course. Using clinical trial simulations, we showed that plasma NfL might be used as an outcome measure of neuronal protection and disease progression, to run MSA trials of feasible duration.

Previous studies on NfL in patients with parkinsonian disorders, including MSA, showed that NfL correlates with disease severity but not disease duration,^10,23^ findings that we replicated in MSA in this study. The lack of association between blood NfL and disease duration indicates that the rate of degeneration of myelinated axons is constant throughout the disease course in the non-Parkinson’s disease parkinsonian disorders. These findings together also suggest that NfL in blood may be useful in clinical trials to detect treatment effects on axonal degeneration.

We established the MSA disease trajectories based on NfL concentrations in plasma across disease stages in conjunction with rigorous clinical, cognitive and imaging profiles. We noted significantly raised NfL in early MSA, which was associated with further increase in UMSARS during disease progression. This is followed by stably elevated NfL levels during manifest disease, with subsequent reductions in advanced disease stages although NfL remained significantly higher than in HC. Most cases in which we identified the NfL decline in the later stages presented with severe disease, as represented by the UMSARS suggesting that the reduction in NfL is less likely to be influenced by slow disease progressors, however, this aspect may reflect a subgroup of slow progressors and warrants more targeted follow-up in larger prospective studies. This pattern of change over time has also been described in patients with frontotemporal dementia^24,25^ and primary progressive aphasia^26^ while in controls a more linear association has been noted.^27^ A sigmoidal pattern of NfL rise of disease course resembles similar findings in Huntington disease.^28^ The NfL trajectory was distinct from that in HC, with little overlap. This suggests that monitoring NfL against an age-relevant reference range derived from the healthy population might be clinically meaningful.

NfL concentrations at a given time point correlated with regional brain volumes and were also indicative of the likely rate of brain atrophy. This association with brain volumes and brain atrophy rates was specifically noted in the striatum, pons volumes and MCP width. A similar correlation was noted in the longitudinal cohort. These areas correlate well with MSA neuropathology as evidence by post-mortem^29^ and neuroimaging studies.^30–34^ The correlation between rate of NfL change and atrophy rate of brain regions suggests that the speed of neuronal breakdown might determine the amount of NfL shed into the extracellular fluid and, ultimately, into the blood. Although rates of change in the NfL had some prognostic value, a single measurement of NfL at baseline showed a stronger ability to predict subsequent clinical decline, brain atrophy and disease state. NfL constitutes a core part of the axonal backbone and is an indicator of degeneration in large myelinated axons.^35^ Some of the weaker correlations with neuroimaging and brain regions could be due to the fact that NfL is tracking secondary axonal damage as the pathology in MSA primarily involves misfolded alpha-synuclein and oligodendroglia. Furthermore, neuronal cell bodies, axons and processes contribute to MRI volumes and could play an important role. Although not yet applied to MSA, a multimodal approach combining volumetric, structural and functional neuroimaging has improved diagnostic accuracy in other neurodegenerative disorders on the basis of structural and functional white matter involvement.^36^ A similar approach could provide significant qualitative and quantitative markers of disease and progression for MSA where the white matter tracts are affected early in the disease course.

Consistent with previous observations^37^, in the group overall, plasma NfL concentration correlated positively with CSF NfL concentration, although this correlation was weak (rho = 0.4). Exploring patients based on disease stages suggested that early MSA cases had a higher-fold change in CSF compared to plasma. The mean fold changes were similar in both biofluids later in the disease. This finding mirrors previous studies in sporadic Alzheimer disease^20,38^ and HD^28^ although several physiological confounding factors that influence the accuracy of blood NfL measurement such as lower concentrations compared to CSF, and degradation and clearance could be possible contributors.^39^ More significant leakage of NfL from the CNS into the blood in later disease, from blood–brain barrier breakdown, may also account for this discrepancy.

We had shown previously that blood NfL differentiates MSA-C from non-MSA sporadic adult-onset ataxia with an area under the curve of 0.74 (95% CI 0.59–0.89), *P* = 0.004.^12^ Similarly, blood NfL can be used to distinguish between patients with Parkinson’s disease and patients with MSA with high diagnostic accuracy (areas under the curve 0.81–0.91) with similar performance for both blood and CSF NfL.^9^

No previous studies have assessed the NfL profile and its prognostic value in large MSA cohorts. We found that higher plasma NfL values at baseline were associated with a more rapid progression and shorter overall survival. Our sample size estimates for future treatment trials aiming to lower NfL blood levels in MSA showed that 14 subjects per study arm would suffice to detect therapeutic effects, even for therapeutic effect sizes as low as 30%. This number is considerably below the cohort sizes, which would probably be required for clinical endpoints, e.g. UMSARS score, estimated at 129 patients per study arm to achieve the same effect size.^40^ NfL levels might therefore provide means for significantly reducing trial sample size. To date, there is no other validated biomarker for MSA that has demonstrated a similarly strong association across a range of clinical, functional and neuroimaging outcomes. Our findings suggest that NfL concentrations in plasma offer an accessible method to evaluate and predict neuronal damage in MSA. Given the rapidly progressive nature of MSA, this is biomarker is high value for assessing the disease outcome. Our study is not without limitations. First, some of the cross-sectional and longitudinal correlations of NfL with existing outcome measures are small, probably due to both biological and measurement variability. Accurate quantification of putaminal atrophy, for example, is particularly challenging. Also, some analysis included duration of illness as a covariate based on first symptoms attributed to MSA, which can be subjective. However, the clinical diagnoses were made by neurologists specialized in MSA and the patients were followed over time with reassessments at each follow-up visit.

For the NfL trajectories in MSA we found a high level of unexplained variability in plasma NfL. Despite narrow within-subject variation making as required for a good biomarker for disease monitoring, there was considerable between-subject variation even in HC.^41^ There is evidence that NfL levels are subject to considerable biological variation include age effects (this aspect was included in our analysis as covariate), but a more detailed analysis of comorbidities could also be included in future work. Recent studies reported confounding effects on NfL values including cardiometabolic risk factors,^42^ vascular comorbidities or renal function,^43^ particularly in older adults. Understanding the sources of the between- and within-subject variation is essential to identify clinically relevant NfL change from other dynamic biological variations.

Second, the CSF cohort was smaller than the cohort of patients with plasma NfL. Therefore, we could not determine whether measurement in plasma is a sufficient alternative or whether there is additional value for paired NfL quantification in CSF, particularly in the early stages of disease. However, as diagnostic certainty in MSA increases with disease severity, we cannot exclude that some patients in early disease stages from our study may turn out to have other parkinsonian diseases or sporadic cerebellar ataxias. Therefore, we show a degree of circumspection when interpreting the results in the early MSA subgroup. Third, we do not yet have longitudinal data on NfL concentrations in CSF. We consider this to be an important aspect as we report a strong association between NfL and disease severity. Furthermore, our MSA disease trajectory analyses were based on baseline clinical and imaging scores. Longitudinal data from this cohort will be essential to accurately characterizing the clinical progression of MSA and identifying markers that predict and/or track progression. To address these issues, and to enable comparison of NfL with other proposed biomarkers, we have set up several multicentre MSA longitudinal studies (PROSPECT-M-UK, BIOPARK, IRAMS and ASPIRE-MSA) assessing biomarkers in association with clinical and neuroimaging outcomes. Finally, we note that overall NfL was a strong predictor of disease severity and progression in this study. However, its variability was too great to allow confident prediction in individuals.

In summary, comprehensive analysis of MSA NfL concentrations in plasma yielded robust results in a multi-site European study of MSA, and provides evidence that NfL is a biomarker of clinical severity, future clinical progression, survival and volumetric brain changes in MSA. We suggest that NfL has a potential role, once validated to regulatory standards, in facilitating the development of novel disease-modifying therapeutics and guiding treatment decisions. We recommend that quantification of NfL concentration in plasma be included in future observational and therapeutic trials for MSA. Retrospective analysis in blood samples collected in previous trials might also be useful, to test for evidence that interventions had effects on neuronal damage, even if the clinical outcomes were negative. Our longitudinal analysis of plasma NfL data, the correlation with validated clinical rating scales and brain structure, together with disease modelling and assessment of sample size estimates for clinical trials, makes the value of plasma NfL more comprehensive for patient stratification and treatment effect monitoring in MSA clinical trials.

## Supplementary Material

awac253_Supplementary_Data
