# Supplementary material for: Neurofilament light levels predict clinical progression and death in multiple system atrophy
Source: Brain. 2022 Jul 29;145(12):4398–408. doi: 10.1093/brain/awac253 (PMC9762941; doi:10.1093/brain/awac253)
Supplement: awac253_Supplementary_Data [file awac253_supplementary_data.pdf]

Supplementary S1. Detailed statistical analysis

Clinical scores

The UMSARS was administered by MSA specialist clinicians. Disease severity was defined by total UMSARS (I+II). Disease duration was defined as time between first reported symptoms considered to be attributable to MSA (onset) and the blood/CSF sample collection. Survival was defined as time between CSF/blood collection and death. Clinically relevant disease milestones were based on previous clinicopathological and MSA natural history studies[1, 2] and were defined as progression to the maximum score in the four UMSARS items (feeding by nasogastric tube or gastrostomy because of severe dysphagia, falls at least once a day, unintelligible speech, and the inability to walk). The following clinical disease milestones were defined: milestone 1 = minimal motor symptoms, score <1 on UMSARS 1 item 7 (walking independently or mildly impaired, no walking aid required), milestone 2 = score of <2 on UMSARS 1 item 7 (walking moderately impaired, assistance and/or walking aid needed occasionally), milestone 3 = score of >3 on UMSARS 1 item 7 (walking severely impaired, assistance and/or walking aid needed frequently or wheelchair-bound), milestone 4 = a score of >3 on UMSARS 1 item 2 (marked impairment of swallowing, frequent food aspiration, nasogastric tube or gastrostomy feeding), and milestone 5 = UMSARS IV, score 5 (dependent in all daily activities).

MRI

In 55 patients, volumetric T1-weighted 3T MR images were acquired within 6 months of blood and CSF collection (mean CSF-MRI interval ±1 month). Of these, 14 patients had a follow-up scan at 12 months (mean interval between scans 13·5±7·0 months).

Similarly, baseline MRI was acquired for the 42 controls matched for age and sex. No follow-up MRI was obtained for controls.

MRI was acquired either on a Trio (Siemens, Erlangen, Germany, TR = 2200 ms, TI = 900 ms, TE = 2.9 ms, acquisition matrix = 256 × 256, spatial resolution = 1·1 mm) or on a Prisma scanner (Siemens, Erlangen, Germany, TR = 2000 ms, TI = 850 ms, TE = 2·93 ms, acquisition matrix = 256 × 256, spatial resolution = 1·1 mm). For each individual included in the longitudinal study, the follow-up scans were acquired on the same scanner as the baseline one.

MR scans were first bias field corrected and whole-brain parcellated using the geodesic information flow (GIF) algorithm,[3] which is based on atlas propagation and label fusion. We extracted the volumes of the following regions: whole brain, striatum (putamen and caudate), pons, and cerebellum (grey and white matter). Left and right volumes were summed, and total intracranial volume (TIV) was computed with SPM12 v6470 (Statistical Parametric Mapping, Wellcome Trust Centre for Neuroimaging, London, UK) running under Matlab R2014b (Math Works, Natick, MA, USA).[4] All segmentations were visually checked for quality. For cross-sectional analysis and baseline values we expressed the volumes as % of TIV. For longitudinal MRI analysis we used the raw volumes. For measuring the middle cerebellar peduncle (MCP) width, bias-corrected MR scans were rigidly registered to the MNI space. We used ITKSnap program (ITK-SNAP 3·8·0-beta) to load the image and used the sagittal view that best exposed the MCP by adapting previously described criteria.[5] We drew a straight line connecting the most ventral point of the pontocerebellar cisterns to the most dorsal point of the cistern. Each MCP (left and right) was measured, and a mean value for the two MCPs was calculated (Supplementary Figure 2).

Group effects

NfL concentrations in plasma and CSF from MSA patients were non-normally distributed, hence a nonparametric analysis was conducted. We used Wilcoxon's rank sum test to compare groups and the Spearman rank correlations to assess associations between the variables. To investigate and adjust the associations with further covariates (age, sex, disease duration), we employed nonparametric kernel regression with 1000 bootstrap replications.[6]

Associations between NfL and disease severity, disease progression and survival

To analyse changes in plasma NfL and UMSARS, we employed linear mixed effects model (LMEM), where the subsequent follow-ups after initial visit at baseline were at 0·5, 1, 2, 3, 3.5, 4 and over 5 years. One level random intercept is included in the model across the patients. No random slop is included in the model. The standard deviation of random intercept of changes of NfL and UMSARS among individuals is not the same and it is significantly different (SD=20.21, CI: 15.93-25.64, p<0.001). Cross-sectional and longitudinal associations were assessed for individual baseline and follow-up measurements in LMEM. Categorical variables are reported as numbers and percentages. To compare the categorical variables, we used the chi-square test.

We used Cox proportional hazard survival modelling to calculate hazard ratios (HRs) with 95% CIs between baseline NfL concentration and subsequent survival. NfL as predictor for survival (after biofluid collection) in patients was analysed using the Log Rank test and Kaplan-Meier curves comparing NfL tertiles and as continuous variables, and a Cox regression with correction for age, both on tertiles and NfL as continuous variables.

Diagnostic accuracy of NfL and cut off values

The accuracy of the diagnostic value of NfL for differentiating MSA cases from HC was assessed by calculating the area under the curve (AUC) of the receiver operating characteristic (ROC) curve. Firstly, we carried out ROC analyses for NfL in a cohort where matched plasma-CSF samples were available in MSA and HC to find the optimal cut off values. The cut-off value of NfL was calculated as the concentration that gave the maximum Youden's index (J = sensitivity + specificity – 1). Then we applied these cut off values to the cohort of MSA cases where we had unpaired plasma samples and determined the specificity and sensitivity for the entire MSA cohort at these cut offs.

Modelling MSA disease trajectory

We used disease duration–by-NfL concentration comparison to generate MSA-specific curves across disease progression. We constructed MSA models of disease duration and plasma NfL concentrations using a two-degree polynomial regression model of main effects and interactions of disease duration and plasma NfL. We used a polynomial model that included linear and squared terms for variables and interactions between them to include higher order terms to ensure that the plateau does not result from the model assumptions but rather from the data. By combining our baseline cross-sectional values from 212 MSA cases, we modelled longitudinal plasma NfL trajectories across disease duration at sample collection range of 0–15 years and adjusted for UMSARS, age at sample collection and sex, to show the time point plasma NfL is predicted to rise, plateau and fall.

Association between absolute NfL levels with neuroimaging and clinical parameters

In cross-sectional (*n*=55) and longitudinal samples (*n*=14, total number of scans = 33), absolute NfL levels were evaluated for association with each of the regional brain volumes, MCP width, UMSARS and MoCA through multiple linear regression, adjusting for age, sex, disease duration and in brain volume analyses, for MRI scanner type. We addressed the issue of multiple collinearities by calculating the variance inflation factor (VIF).

Calculating the rate of change in biomarkers

Rates of change were extracted from LMEM, using time (in years) as a fixed effect. Random slopes and intercepts of time per participant were included to produce individual regression coefficients per participant as previously described.[7] The rate of NfL change per individual was extracted from the model estimates for subsequent analyses. This model was also used for generating the rate of change for regional brain volumes and MCP width, UMSARS and MoCA.

Relating NfL rate of change to imaging and UMSARS rate of change

We determined the longitudinal relationship between the rate of change in plasma NfL and concurrent rate of change in the UMSARS, brain volumes of interest and the MCP width using LMEMs. The dependent term for each model was an imaging biomarker and UMSARS with fixed effect terms for baseline age, sex, time from baseline, extracted rate of change in plasma NfL, and interaction between time from baseline and rate of change in plasma NfL. Models contained random slope and intercept terms for participants. The primary term of interest was the interaction between the rate of change in plasma NfL and the time from baseline term. Models were fitted using lme4 in R.

Prospective prediction of brain volumes and UMSARS by plasma NfL rate of change

To investigate whether baseline plasma NfL could predict subsequent yearly changes in regional brain volume, cognition, and UMSARS scores we conducted a prospective study whereby following longitudinal plasma collection for NfL, we collected additional imaging and UMSARS data on 14 MSA cases.

To determine the rate of change in brain volumes and UMSARS between the imaging and clinical assessment concurrent to the participant's last blood draw and follow-up session, we fitted an LMEM for each participant, where the dependent variable was the imaging or UMSARS variable of interest at last plasma visit and follow-up visit, and the independent variable was the time between visits. We then used this rate of change for brain regions or UMSARS as the dependent variable in an LMEM, which included fixed effects for age, sex, and the rate of change in plasma NfL, and a random intercept term participant. The term of interest was the plasma NfL rate of change. Models were fitted using lme4 in R.

Sample size estimation for intervention trials

We derived the sample size per arm required to inform the design of therapeutic trials aiming to lower NfL by a range of desired therapeutic effect sizes. Given that the NfL changes in our cohort showed non-linear trajectories over time, the estimation assumed a non-linear progression. The estimation further assumed equal numbers of subjects in both study arms (allocation ratio 1:1), $\alpha=0.05$, $\beta=0.01$, two-tailed independent t-tests, and the use of plasma NfL levels. Based on these assumptions, we derived the sample size per arm required to detect a given control-adjusted percent reduction in the treatment arm with 80% or 90% power and two-sided 5% type I error.

Supplementary Figure 1. Project set-up and neuroimaging measurements of the brain regions of interest.

A

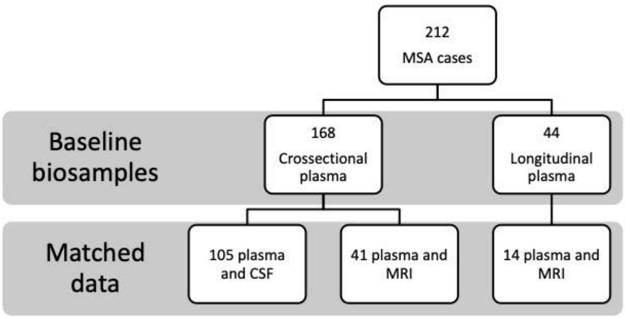

B

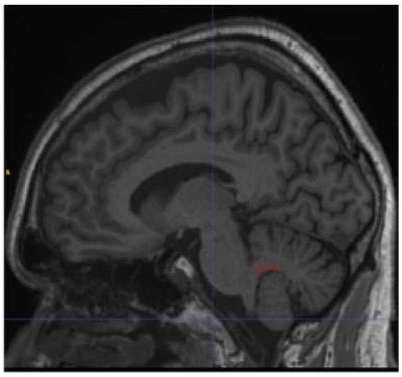

A.Flow-chart of sample and imaging data collected for this study. **B.** Example of the measuring of the middle cerebellar peduncle using volumetric T1-weighted MRI on scans realigned on the MNI space.

Supplementary Figure 2. Correlation analysis of NfL concentration measured in plasma and CSF.

A. NfL levels in plasma and CSF were moderately correlated in the MSA cohort (*rho*=0.40, $p<0.001$) and (**B**) highly correlated in the healthy control group (*rho*=0.7, $p<0.001$) using Spearman rank-order correlation test. NfL = neurofilament light chain. **C.** CSF and plasma NfL concentrations in different diagnostic groups stratified by disease duration. The concentrations of NfL in plasma and CSF were measured in 105 cases where matched plasma and CSF was available. The MSA cases were stratified by disease duration in early disease (less than three years of disease duration), established

disease (between 3-7 years) and late disease (more than 7 years of disease duration). Correlation analysis was performed using Spearman’s rank-ordered correlation coefficient. **D.** Comparison of fold-changes in mean NfL CSF and NfL plasma between cases and healthy controls stratified by disease duration, as above, where matched CSF and plasma NfL data was available. **E.** Plasma NfL concentration in patients with MSA and diagnostic accuracy. Receiver operating characteristic (ROC) curves showed high accuracy for both CSF (blue line) and plasma NfL (red line) in MSA (AUC=0.985; 95% CI 0.803 to 0.912 for CSF AUC=0.858; 95% CI 0.8 to 0.9 for plasma). ROC curves showed high accuracy of plasma NfL in distinguishing between MSA cases and healthy controls (AUC=0.996, with a cut-off at 10.6 pg/mL to guarantee maximum sensitivity (99%) and specificity (89%). AUC=area under the curve; NfL=neurofilament light chain, CSF= cerebrospinal fluid; MSA=multiple system atrophy.

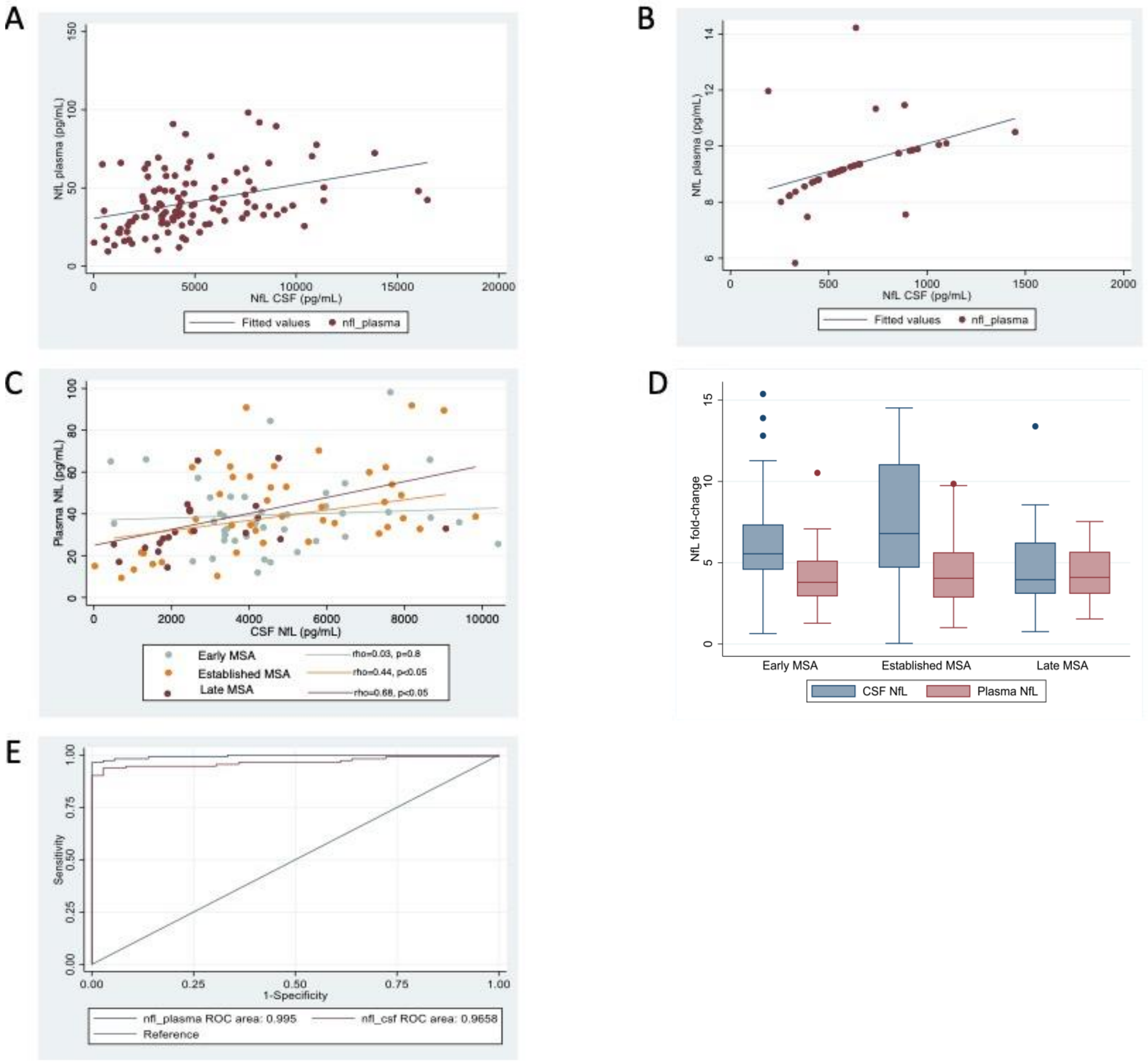
A
NfL plasma (pg/mL)
NfL CSF (pg/mL)
Fitted values
nfl_plasma
B
NfL plasma (pg/mL)
NfL CSF (pg/mL)
Fitted values
nfl_plasma
C
Plasma NfL (pg/mL)
CSF NfL (pg/mL)
Early MSA
Established MSA
Late MSA
rho=0.03, p=0.8
rho=0.44, p<0.05
rho=0.68, p<0.05
D
NfL fold-change
Early MSA
Established MSA
Late MSA
CSF NfL
Plasma NfL
E
Sensitivity
1-Specificity
nfl_plasma ROC area: 0.995
nfl_csf ROC area: 0.9658
Reference

Supplementary Figure 3. Analysis of plasma NfL, survival in early stage MSA and longitudinal dynamics. A. Receiver operating characteristic (ROC) curves showed high accuracy for plasma NfL in distinguishing early stages MSA from controls. The area under the curve (AUC) is 0.999 and plasma NfL cut-off 12 pg/mL offers 98.6% sensitivity and 94.4% specificity. **C.** Survival distribution of 210 MSA patients based on plasma NfL tertile value. Lower plasma NfL tertile was associated with better survival in MSA patients. 14.3% (10/70) of deaths occurred amongst the group with the lowest plasma NfL tertile values, 32.9% (23/70) of deaths occurred amongst the group with the middle plasma NfL tertiles and 44.3% (31/70) of deaths occurred amongst the group with the highest plasma NfL tertiles. The survival distributions for the three groups were statistically significantly different, χ^2 (2) = 23.401, P <0.001. Red: lowest tertile (9.0 – 30 pg/mL); Green: middle tertile (31 – 46 pg/mL); Blue: Highest tertile. (47 – 119.0 pg/mL), 'Censored' (vertical ticks) refer to living patients. **C.** Spaghetti plot showing longitudinal plasma NfL for changes (n=44) using a multilevel analysis (p=<0.001) as a function of disease duration and subsequent increase in UMSARS. **D**. To investigate whether baseline plasma NfL could predict yearly changes in brain region volumes and UMSARS scores, linear mixed-effects models were fitted with fixed effect terms for baseline age (age at onset), sex, time, baseline plasma NfL, and an interaction between time and baseline plasma NfL. Random slopes and intercepts of time per participant were included to produce individual regression coefficients per participant, as previously described[7].

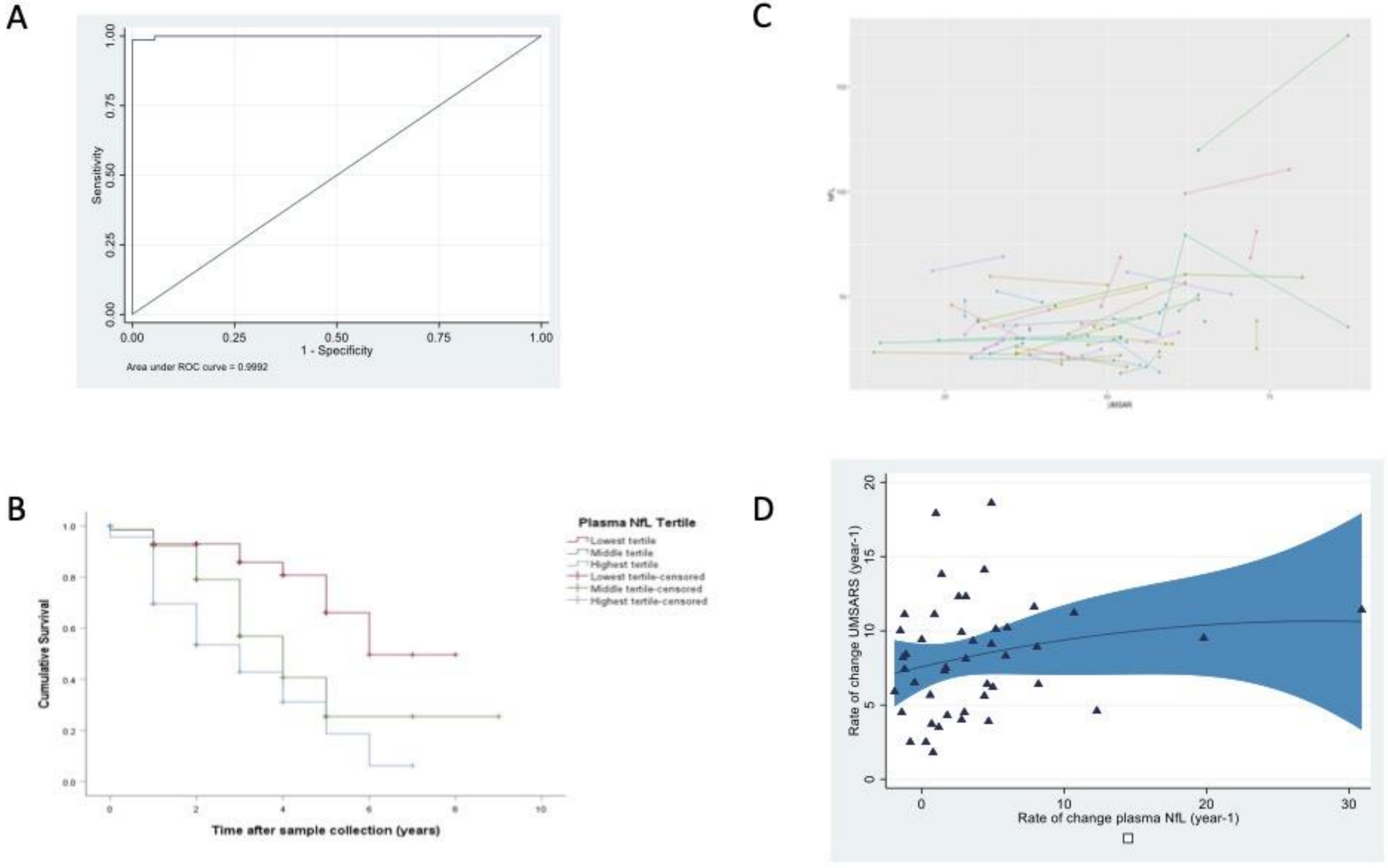
A
Sensitivity
1 - Specificity
0.00
0.25
0.50
0.75
1.00
Area under ROC curve = 0.9992
C
B
Plasma NfL Tertile
Lowest tertile
Middle tertile
Highest tertile
Lowest tertile-censored
Middle tertile-censored
Highest tertile-censored
Cumulative Survival
Time after sample collection (years)
D
Rate of change UMSARS (year-1)
Rate of change plasma NfL (year-1)

References

1. O'Sullivan SS, Massey LA, Williams DR, et al. Clinical outcomes of progressive supranuclear palsy and multiple system atrophy. *Brain*. May 2008;131(Pt 5):1362-72. doi:10.1093/brain/awn065
2. Wenning GK, Geser F, Krismer F, et al. The natural history of multiple system atrophy: a prospective European cohort study. *Lancet Neurol*. Mar 2013;12(3):264-74. doi:10.1016/s1474-4422(12)70327-7
3. Cardoso MJ, Modat M, Wolz R, et al. Geodesic Information Flows: Spatially-Variant Graphs and Their Application to Segmentation and Fusion. *IEEE transactions on medical imaging*. Sep 2015;34(9):1976-88. doi:10.1109/tmi.2015.2418298
4. Malone IB, Leung KK, Clegg S, et al. Accurate automatic estimation of total intracranial volume: a nuisance variable with less nuisance. *NeuroImage*. Jan 1 2015;104:366-72. doi:10.1016/j.neuroimage.2014.09.034
5. Nicoletti G, Fera F, Condino F, et al. MR imaging of middle cerebellar peduncle width: differentiation of multiple system atrophy from Parkinson disease. *Radiology*. Jun 2006;239(3):825-30. doi:10.1148/radiol.2393050459
6. Byrne LM, Rodrigues FB, Blennow K, et al. Neurofilament light protein in blood as a potential biomarker of neurodegeneration in Huntington's disease: a retrospective cohort analysis. *The Lancet Neurology*. Aug 2017;16(8):601-609. doi:10.1016/s1474-4422(17)30124-2
7. Preische O, Schultz SA, Apel A, et al. Serum neurofilament dynamics predicts neurodegeneration and clinical progression in presymptomatic Alzheimer's disease. *Nature medicine*. Feb 2019;25(2):277-283. doi:10.1038/s41591-018-0304-3
